# Characterizing Plasma Biomarkers of Alzheimer's in a Diverse Community-Based Cohort: A Cross-Sectional Study of the HAB-HD Cohort

**DOI:** 10.3389/fneur.2022.871947

**Published:** 2022-08-18

**Authors:** James R. Hall, Melissa Petersen, Leigh Johnson, Sid E. O'Bryant

**Affiliations:** ^1^Institute for Translational Research, University of North Texas Health Science Center, Fort Worth, TX, United States; ^2^Department of Family Medicine, University of North Texas Health Science Center, Fort Worth, TX, United States

**Keywords:** Alzheimer's, plasma, biomarker, diverse populations, mild cognitive impairment (MCI)

## Abstract

**Background:**

Due to their low cost, less invasive nature, and ready availability, plasma biomarkers of Alzheimer's disease have been proposed as one-time screening tools for clinical trials and research. The impact of ethnoracial factors on these biomarkers has received little attention. The current cross-sectional study investigated the levels of Aβ_40_, Aβ_42_, total tau (t tau), and neurofilament light (NfL) across diagnoses for each of the three major ethnoracial groups in the United States in a community-based cohort of older adults.

**Methods:**

A total of 1,862 participants (852 Mexican Americans (MAs); 775 non-Hispanic Whites (NHWs), and 235 African Americans (AAs)) drawn from The Health & Aging Brain Study—Health Disparities (HABS-HD) study were included. Diagnoses were assigned using an algorithm (decision tree) verified by consensus review. Plasma samples were assayed using Simoa technology. Levels of each biomarker were compared for the three ethnoracial groups across cognitive diagnoses using ANOVA covarying sex and age.

**Results:**

Significant differences were found across the groups at each level of cognitive impairment. Cognitively unimpaired (CU) AA had significantly lower levels of each of the biomarkers than cognitively unimpaired MA or NHW and NHW had higher levels of Aβ_40_, and NfL than the other two groups. MA had higher t tau than AA or NHW. Mild cognitive impairment (MCI) group NHW had the highest levels on all the biomarkers and AA had the lowest. NHW and MA have higher levels of Aβ_40_, Aβ_42_, and t tau there was no difference between the groups for Aβ_42_. NHW had significantly higher levels of Aβ_40_, t tau, and NfL than AA. AA had a higher Aβ_42_/Aβ_40_ ratio than either NHW or MA for CU MCI.

**Conclusions:**

The use of plasma biomarkers of cognitive decline is promising given their advantages over other biomarkers such as CSF and imaging but as the current research shows, ethnoracial differences must be considered to enhance accuracy and utility. Developing ethnoracial-specific cut points and establishing normative ranges by assay platform for each of the biomarkers are needed. Longitudinal research to assess changes in biomarkers during a cognitive decline is ongoing.

## Introduction

Recently there has been a significant increase in research related to the utility of plasma-based biomarkers of Alzheimer's disease (AD) as cost-effective, minimally invasive, and highly scalable tools to aid in clinical practice and clinical trials (1–6). Although there is no indication that these plasma-based biomarkers individually or as a group can be used as “diagnostic,” their use has the potential to aid in determining who may need more invasive and costlier confirmatory diagnostic procedures such as lumbar punctures or positron emission tomography (PET) scans (7–10). The plasma biomarkers of amyloid-beta (Aβ_40_, Aβ_42_, total tau (t tau), and neurofilament light (NfL)) have been shown to correlate with cognitive decline and brain atrophy (11). Lower ratios of Aβ_42_/Aβ_4_ have been linked to brain amyloidosis (12, 13) and increased risk for developing cognitive impairment (14, 15). Despite the surge in research on plasma AD biomarkers of amyloid (Aβ_40_ and Aβ42) and neuronal degeneration (t tau and NfL), there remain significant gaps in our understanding of the basic functioning of these biomarkers and the factors that may influence their levels. The majority of research has been conducted in clinical trials or clinical research settings using CSF biomarkers of AD and the vast majority of participants have been non-Hispanic Whites (NHWs). Although Hispanic and African American (AA) communities have significantly higher rates of dementia (16) and therefore may gain significant benefit from the use of AD biomarkers in clinical assessment, there is limited research on blood-based biomarkers of AD in these communities (4, 17–20).

Of the studies that have been conducted on plasma biomarkers and ethnicity, most have been bi-ethnic/biracial studies comparing Hispanics or AA to NHWs across diagnostic groups or research on one of the ethnoracial groups comparing diagnostic groups. Gonzales et al. (21) found that t tau and NfL discriminated between diagnostic groups for both Hispanics (predominately Mexican Americans (MA)) and NHWs. The same study found no difference in NfL levels in a demographically matched subset of the two ethnic groups. O'Bryant et al. (4) in a bi-ethnic study of MAs and NHWs found NfL significantly associated with diagnostic groups for both ethnic groups. A study of a diverse Florida sample found no effect for Hispanic ethnicity on NfL levels (22). In a study of community-based older MAs, NfL levels were significantly higher for cognitively impaired [mild cognitive impairment (MCI) and dementia] participants compared to cognitively unimpaired (23).

A Washington Heights and Inwood Community Aging project community-based study of plasma biomarkers found no significant differences across Hispanic, Black, and NHWs in concentrations of t tau, Aβ_40_, Aβ_42_, or NfL (24). Gerwal et al. (25) in a small study of women (*N* = 15 in each group) with MCI reported higher levels of Aβ_40_ and Aβ_42_ in Hispanic women compared to AA and NHW women. A study of plasma biomarkers in AAs including tau and Aβ_42_ comparing cognitively unimpaired individuals with Alzheimer's patients found that tau was significantly higher in the AD group and Aβ_42_ level was not associated with Alzheimer's (26). A discovery-based study of plasma comparing AAs to NHWs demonstrated the importance of including diverse racial and ethnic groups in the development of effective biomarkers (27).

The current study was conducted to investigate the impact of ethnicity/race on the level of the plasma biomarkers of Aβ_40_, Aβ_42_, t tau, and NfL the three largest ethnic/racial groups in the United States, in a community-based sample of MAs, AAs, and NHWs.

## Methods

### Participants and Assessment

This study included 1,862 participants (852 MAs; 775 NHWs; and 235 AAs) drawn from The Health & Aging Brain Study—Health Disparities (HABS-HD) study. The HABS-HD study is an ongoing, longitudinal, project examining health disparities in cognitive aging among community-dwelling older MAs. The study cohort was initially composed of MAs and NHWs Click or tap here to enter text.and was recently expanded to include AAs (28). The goal of the study is to recruit 1,000 participants for each group. The HABS-HD methods have been described in detail elsewhere (7). Briefly, the HABS-HD protocol includes an interview, functional exam, blood draw for clinical labs and biobanking, neuropsychological testing, and 3T MRI of the brain. All aspects of the study protocol can be conducted in Spanish or English based on the preference of the participant. A study partner with knowledge of the participant is interviewed for clinician completion of the clinical dementia rating amyloid and tau PET scans are ongoing for the full cohort.

Inclusion criteria include (1) self-identified ethnicity/race of AA, MA, or NHW, (2) willingness to provide blood samples, (3) capable of undergoing neuroimaging studies, (4) age 50 and above, and (5) fluent in English or Spanish. Exclusion criteria are (1) type 1 diabetes, (2) presence of active infection, (3) current/recent (12 months) cancer (other than skin cancer), (4) current severe mental illness that could impact cognition (other than depression), (5) recent (12 months) traumatic brain injury with loss of consciousness, (6) current/recent alcohol/substance abuse, (7) active severe medical condition that could impact cognition such as end-stage renal failure, chronic heart failure, or chronic obstructive pulmonary disease, and (8) current diagnosis of non-Alzheimer's-related dementia.

The HABS-HD study is conducted under IRB-approved protocols and each participant (or his/her legal representative) signs written informed consent. The data are available to the scientific community through the UNTHSC Institute for Translational Research (ITR) website (29).

### Diagnostic Classification

#### Diagnoses

Cognitive diagnoses are assigned using an algorithm (decision tree) that is verified at consensus review by a panel of experienced Alzheimer's clinicians. Cognitively unimpaired (CU) = no cognitive complaints, CDR sum of boxes score of 0, and cognitive test scores broadly within normal limits (i.e., performance no more than 1.5 SDs below the mean of the normative range on any test]); MCI: cognitive complaint (self or other), CDR sum of boxes score between 0.5 and 2.0 and at least one cognitive test score falling < =1.5 SD below normative ranges. Dementia: CDR sum of boxes score >=2.5 and at least two cognitive test scores 2 SD below normative ranges.

### Assays

#### Blood Collection and Processing Procedures

Samples were assayed in the University of North Texas Health Science Center Institute for Translational Research (ITR) Laboratory by the ITR Biomarker Core. Fasting blood collection and processing follow the international guidelines for AD biomarker studies (30). Blood samples were processed within 2 h (stick-to-freezer). Proteomic assays for this study were processed on a multiplex biomarker assay platform using electrochemiluminescence (ECL) using commercially available kits Quanterix. The ITR Biomarker Core has extensive experience utilizing automated systems to assay blood samples and recently reported the analytic performance of each of these markers for *n* > 1,300 samples across multiple cohorts for normal cognition, mild cognitive impairment, and AD. The assays have been shown to be reliable and have excellent spiked recovery, dilution linearity, coefficients of variation (CVs), and detection limits. Very acceptable measures of inter- and intra-assay variability have been found. Internal QA/QC protocols along with manufacturing protocols including assaying consistent controls across batches and assay of pooled standards across lots were implemented.

### Samples

A total of 500 μl of plasma was utilized to measure biomarker levels using the Single Molecule Array (Simoa) technology (Simoa; Quanterix, Lexington, MA, USA). From the materials provided, a recombinant NfL calibration curve was constructed. The calibration range was 0–500 pg/ml with a dynamic range of 0–2,000 pg/ml. The sample and control concentrations were calculated from 4PL curve fit. CV for NfL was 0.038 and Lowest Level of Detection (LLOD) was 0.038 pg/ml.

Utilizing Simoa technology, multiplexed detection of Aβ_42_, Aβ_40_, and t tau was conducted. Calibration ranges for Aβ_42_, Aβ_40_, and t tau were 0–60, 0–140, 0–100 pg/ml and dynamic ranges of 0–240, 0–560, 0–400 pg/ml, respectively. Aβ_42_, Aβ_40_, and t tau control samples (analog 87.0, 393, 99.5 pg/ml and digital 3.20, 22.4, 2.24 pg/ml, respectively). The sample and control concentrations were calculated from a 4PL curve fit. CVs for Aβ_42_, Aβ_40_, and t tau were 0.043, 0.043, and 0.061, respectively. LLODs for Aβ_42_, Aβ_40_, and t tau were 0.045, 0.196, and 0.019 pg/ml, respectively. Interplate CVs were derived for high and low pooled controls from the Quanterix automated system: NfL (high control CV = 0.035 and low control CV = 0.092); Aβ_42_ (high control CV = 0.051 and low control CV = 0.040); Aβ_40_ (high control CV =0.050 and low control CV = 0.042); and t tau (high control CV = 0.040 and low control CV = 0.047).

### Statistical Analysis

The data were analyzed using SPSS-25 (IBM). Group differences were assessed using ANOVA for continuous data and chi-squared for categorical data. Levels of the plasma biomarkers within groups by diagnosis and across groups by diagnoses were analyzed with ANOVA co-varying age and gender. Tukey's honestly significant difference was used to analyze differences between the groups. Statistical significance was set at *p* < 0.05.

## Results

Table 1 presents the characteristics of the sample including select medical comorbities. For the entire sample there was a significant difference in the age of the groups (*F* (92, 1,859) = 128.935, *p* = 0.0000) with the NHW being significantly older than both the AA (Diff = 6.21, *p* = 0.0000) and MA (Diff = 6.23, *p* = 0.0000). There was no significant difference in age between the AA and MA groups (Diff = 0.02, *p* = 0.9948). There was a significant difference in years of education for the groups (*F* (2.1859) = 685.165, *p* = 0.0000) with AA and NHW having significantly more years of education than the MA (Diff = 6.12, *p* = 0.0000 and Diff = 6.32, *p* = 0.0000). There was no difference in years of education between AA and NHW (Diff = 0.20, *p* = 0.7239). The groups did not differ in the distribution of the sexes. Among the medical comorbities, there was a significant difference across the groups in rates of hypertension, dyslipidemia, and diabetes with NHW having the lowest rates for all three disorders. There was no significant difference between the groups in rates of depression or anxiety.

**Table 1:** Characteristics of the sample.

	**Non-Hispanic White** ***N* = 775**	**Mexican Americans** ***N* = 852**	**African American/Blacks** ***N* = 235**	
Age	M = 69.30 SD = 8.68	M = 63.07 SD = 7.98	M = 63.09 SD = 7.71	F (2.1859) = 128.935 *p* = 0.0000*
Education	M = 15.49 SD = 2.57	M = 9.17 SD = 4.59	M = 15.29 SD = 2.65	*F* (2.1859) = 685.165 *p* = 0.0000*
% Female	442 (57%)	571 (67%)	167 (71%)	X^2^ = 4.57 *P* = 0.101
Hypertension	457 (59%)	561 (66%)	181 (77%)	X^2^ = 27.077 *P* = 0.0000*
Dyslipidemia	469 (40%)	572 (67%)	149 (63%)	*X*^2^ = 23.360 *P* = 0.0000*
Diabetes	98 (13%)	313 (37%)	58 (25%)	*X*^2^ = 125.043 *P* = 0.0000*
Depression	254 (33%)	284 (33%)	77 (33%)	X^2^ = 0.066 *P* = 0.9676
Anxiety	124 (16%)	131 (15%)	46 (20%)	X^2^ = 2.437 *P* = 0.2977

*p ≤ 0.05.

Differences in the levels of each of the biomarkers across the three ethnoracial groups by diagnostic category were analyzed using ANOVA covarying sex and age. Table 2 compares the level of the biomarkers across groups for the cognitively unimpaired. There was a significant difference between the groups for each of the biomarkers. NHW had significantly higher levels of Aβ_40_ and NfL than the other two groups. AA had the lowest level for each of the four biomarkers and had the highest Aβ_42_/Aβ_4_ ratio. For Aβ_40_ there was a significant difference between NHW and MA (Diff= 28.287, *p* = 0.0000) and NHW and AA (Diff = 102.634, *p* = 0.0000). MA had a significantly higher level of Aβ_40_ than AA (Diff = 74.347, *p* = 0.0000). For Aβ_42_ there was no difference between NHW and MA (Diff = 0.383, *p* = 0.0832) however NHW and MA had significantly higher levels than AA (Diff = 3.367, *p* = 0.0000 and Diff = 2.984, *p* = 0.0000). Findings for the Aβ_42_/Aβ_40_ ratio showed that MA had a significantly higher ratio than NHW (Diff = 0.0041, *p* = 0.0004) and that AA had a significantly higher Aβ_42_/Aβ_40_ ratio than either NHW or MA. MA had a significantly higher level of t tau than NHW (Diff = 0.2440, *p* = 0.0001) and AA (Diff = 0.8450, *p* = 0.0000). NHW had a significantly higher level of t tau than AA (Diff = 0.6010, *p* = 0.0000). For levels of NfL among the cognitively unimpaired, NHW had higher levels than either MA (Diff = 3.353, *p* = 0.0000) or AA (Diff = 7.521, *p* = 0.0000) and MA had higher levels than AA (Diff = 4.168, *p* = 0.0011).

**Table 2:** Normal cognition.

	**Non-Hispanic Whites** ***N* = 644**	**Mexican Americans** ***N* = 642**	**African Americans *N* = 142**	***F* Statistic**
Aβ_40_	M = 266.014 SD = 62.656 95% CI [261.167, 270.861]	M = 237.727 SD = 66.410 95% CI [232.581, 242.872]	M = 163.380 SD = 41.831 95% CI [156.463, 170.297]	*F* (2.1425) = 160.258 *P* = 0.0000*
Aβ_42_	M = 12.238 SD = 3.121 95% CI [11.996, 12.480]	M = 11.855 SD = 3.350 95% CI [11.595, 12.115]	M = 8.871 SD = 3.027 95% CI [8.370, 9.371]	*F* (2.1425) = 64.585 *p* = 0.0000*
Tau	M = 2.311 SD = 1.066 95% CI [2.229, 2.392]	M = 2.555 SD = 1.065 95% CI [2.472, 2.639]	M = 1.710 SD = 0.644 95% CI [1.604, 1.816]	*F* (2.1425) = 40.3912 *p* = 0.0000*
NfL	M = 20.100 SD = 12.689 95% CI [19.119, 21.080]	M = 16.747 SD = 12.968 95% CI [15.744, 17.749]	M = 12.579 SD = 10.451 95% CI [10.850, 14.307]	F (2.1425) = 25.098 *p* = 0.0000*
Aβ_42_/Aβ_40_	M = 0.0473 SD = 0.0133 95% CI [0.046, 0.048]	M = 0.0514 SD = 0.0217 95% CI [0.050, 0.053]	M = 0.0651 SD = 0.0289 95% CI [0.063, 0.067]	*F* (2.1425) = 49.168 *P* = 0.0000*

*p ≤ 0.05.

Table 3 presents the levels of each of the biomarkers for individuals diagnosed with MCI. NHW had the highest levels of all the biomarkers and AA had the lowest. NHW had significantly higher levels of Aβ_40_ than MA (Diff = 33.685, *p* = 0.0002) and AA (Diff = 109.873, *p* = 0.0000). MA had a significantly higher level of Aβ_40_ than AA (Diff = 76.188, *p* = 0.0000. The level of Aβ_42_ did not differ between NHW and MA (Diff = 0.5020, *p* = 0.5093). Both NHW and MA had significantly higher levels of Aβ_42_ than AA (Diff = 2.9590, *p* = 0.0000 and Diff = 2.4570, *p* = 0.0000). For the Aβ_42_/Aβ_40_ ratio, there was no difference between NHW and MA (Diff = 0.0049, *p* = 0.1143). AA had a significantly higher Aβ_42_/Aβ_40_ ratio than either NHW (Diff = 0.0117, *p* = 0.0002) or MA (Diff = 0.0068, *p* = 0.0235). The level of t tau did not differ between NHW and MA (Diff = −0.0380, *p* = 0.9664) and both NHW and MA had significantly higher levels of t tau than AA (Diff = 0.9520, *p* = 0.0000, and Diff = 0.9900, *p* = 0.0000). NHW had significantly higher levels of NfL than either MA (7.479, *p* = 0.0000) or AA (Diff = 11.325). MA and AA did not differ on NfL level (Diff = 3.846, *p* = 0.0747).

**Table 3:** MCI.

	**Non-Hispanic Whites** ***N* = 86**	**Mexican Americans** ***N* = 146**	**African Americans** ***N* = 74**	***F* Statistic**
Aβ_40_	M = 278.418 SD = 63.377 95% CI [265.003, 291.833]	M = 244.733 SD = 66.469 95% CI [233.935, 255.530]	M = 168.545 SD = 43.919 95% CI [158.463, 170.297]	*F* (2.303) = 67.809 *P* = 0.0000*
Aβ_42_	M = 12.744 SD = 3.16 2 95% CI [12.074, 13.414]	M = 12.242 SD = 3.347 95% CI [11.697, 12.786]	M = 9.785 SD= 3.485 95% CI [9.098, 10.471]	*F* (2.303) = 18.283 *P* = 0.0000*
Tau	M = 2.624 SD = 1.066 95% CI [2.398, 2.850]	M = 2.662 SD = 1.075 95% CI [2.488, 2.837]	M = 1.672 SD = 1.273 95% CI [1.527, 1.818]	*F* (2.303) = 21.198 *P* = 0.0000*
NfL	M = 25.838 SD = 12.788 95% CI [23.130, 28. 546]	M = 18.359 SD = 12.929 95% CI [16.260, 20.459]	M = 14.513 SD = 10.349 95% CI [12.142, 16.885]	*F* (2.303) = 18.083 *P* = 0.0000*
Aβ_42_/Aβ_40_	M = 0.0465 SD = 0.0109 95% CI [0.044, 0.050]	M = 0.0514 SD = 0.0171 95% CI [0.049, 0.054]	M = 0.0582 SD = 0.0187 95% CI [0.052, 0.059]	*F* (2.303) = 8.396 *P* = 0.0003*

*p ≤ 0.05.

The levels of the four biomarkers for those diagnosed with dementia are presented in Table 4. For Aβ_40_ levels, there was no difference between NHW and MA (Diff = 17.598, *p* = 0.3290) although NHW had significantly higher levels than AA (Diff = 87.851, *p* = 0.0000) and MA had a higher level than AA (Diff = 70.258, *p* = 0.0001). There were no significant differences between the groups on Aβ_42_ levels (NHW vs. MA Diff = 0.642, *p* = 0.5801; NHW vs. AA Diff = 1.790, *p* = 0.1222; and MA vs. AA Diff = 1.148, *p* = 0.3826). There was no difference in Aβ_42_/Aβ_40_ ratio between MA and NHW (Diff = 0.0041, *p* = 0.5986). There was no significant difference between the Aβ_42_/Aβ_40_ ratio for AA compared to MA (Diff = 0.0089, *p* = 0.1469) although AA had a significantly higher ratio than NHW (Diff = 0.0123, *p* = 0.0373). T tau levels significantly differed between NHW and AA (Diff = 0.6070, *p* = 0.0269) along with MA and AA (Diff = 0.7370, *p* = 0.0033). There was no difference between NHW and MA (Diff = −0.1300, *p* = 0.7115). NHW and MA did not differ in the level of NfL (Diff = −0.1300, *p* = 0.9309) but NHW had higher levels of NfL than AA (Diff = 4.132, *p* = 0.0000) as did MA (MA vs AA Diff = 4.2620, *p* = 0.0000).

**Table 4:** Dementia.

	**Non-Hispanic Whites** ***N* = 45**	**Mexican Americans** ***N* = 64**	**African Americans** ***N* = 19**	**F Statistic**
Aβ_40_	M = 262.641 SD = 62.112 95% CI [244.464, 280.818]	M = 245.043 SD = 67.464 95% CI [228.491, 261.595]	M = 174.790 SD = 49.555 95% CI [155.261, 194.318]	*F* (2.125) = 13.158 *P* = 0.0000*
Aβ_42_	M = 11.923 SD = 3.099 95% CI [11.015, 12.830]	M = 11.281 SD = 3.400 95% CI [10.446, 12.116]	M = 10.133 SD = 3.485 95% CI [8.760, 11.507]	*F* (2.125) = 1.9174 *P* = 0.1436*
Tau	M = 2.634 SD = 1.047 95% CI [2.328, 2.940]	M = 2.764 SD = 0.712 95% CI [2.496, 3.032]	M = 2.027 SD = 0.740 95% CI [1.736, 2.317]	*F* (2.125) = 5.555 *P* = 0.0049*
NfL	M = 25.981 SD = 1.869 95% CI [23.311, 30.650]	M = 26.111 SD = 1.651 95% CI [22.870, 29.352]	M = 21.849 SD = 2.407 95% CI [17.106, 26.592]	*F* (2.125) = 41.742 *P* = 0.0000*
Aβ_42_/Aβ_40_	M = 0.0457 SD = 0.0109 95% CI [0.042, 0.050]	M = 0.0491 SD = 0.0184 95% CI [0.045, 0.052]	M = 0.0580 SD = 0.0281 95% CI [0.054, 0.062]	*F* (2.125) = 3.102 *P* = 0.0485*

*P ≤ 0.05.

## Discussion

This study investigates the impact of ethnoracial factors on plasma biomarkers of AD. The data clearly show that the level of many of the biomarkers differ by ethnicity/race and differ by diagnosis. Regardless of diagnosis, the levels of all four plasma biomarkers for AA were significantly lower than the NHW. MA had higher levels than AA of all the biomarkers in both the cognitively unimpaired and MCI diagnostic groups. Whereas, NHW had significantly higher levels of Aβ_40_ and NfL than MA in the cognitively unimpaired and MCI groups. MA with normal cognition had a significantly higher level of t tau than NHW, although there was no difference between the two groups on tau level in the impaired groups. NHW across all diagnoses had the lowest Aβ_42_/Aβ_4_ ratio with AA having a significantly higher ratio than NHW. These findings support the importance of ethnicity/race in any study utilizing these plasma biomarkers as categorizing or outcome variables.

Although the overall sample size was robust and community-based, there are several limitations that affect the generalizability of the findings. We utilized clinical rather than imaging biomarker-based criteria for the diagnostic assignment that may have had an impact on the validity of our diagnoses. The problem of diagnostic validity will be resolved in the ongoing study as the entire HABS-HD cohort is currently undergoing brain amyloid scans. Another weakness is the cross-sectional nature of the data; however, HABS-HD is a longitudinal study and follow-up studies to assess changes in plasma biomarkers over time will be conducted in the future. The size of the AA sample relative to the other two groups is somewhat problematic especially given the small number of AA dementia cases. The goal of HABS-HD is to recruit 1,000 NHW, 1,000 MA, and 1,000 AA participants. The cohort was initially established as a study of MA cognitive aging with NHWs as a comparison group. Recently, the recruitment of an AA sample was initiated and the sample size reflects the current level of recruitment. The age difference between NHW and MA and AA groups may have an impact on the results and this variable needs to be directly addressed in further research. It is important to note that the AA and MA groups did not differ in age and the differences in biomarkers between these groups are likely not attributable to age. Recent studies have indicated that medical comorbidities such as chronic kidney disease (31, 32) can affect levels of plasma biomarkers of amyloid and neurodegeneration. The exclusion criteria for the study attempt to limit the effect of specific medical conditions on cognition but this may not adequately account for the impact of medical comorbidities on the level of these biomarkers. Even with these limitations, given the current efforts to apply one-time plasma AD biomarkers as screening tools and diagnostic markers, the current findings have direct applicability to these efforts.

The current findings also point to the difficulty of developing standard cut points for the biomarkers given the ethnoracial differences. In addition, the development of appropriate normative values for the biomarkers specific to each of the groups would require relatively large samples of well-characterized ethnoracial groups. There is also the issue of the variety of assay platforms that can produce results that are not comparable. Even when the same platform is used, sample characteristics can produce different results. For example, a study of the Rotterdam (33) cohort used the same platform as our study to assay tau, NfL, Aβ_40_, and Aβ_42_ in a cognitively unimpaired, overwhelming white population. The level of Aβ_42_, tau, and Nfl we found in our cognitively unimpaired NHW were significantly higher than those found in the Rotterdam study. When comparing the biomarker level for AA with a Mayo study of AA (26) that also used the Quanterix Simoa platform both Aβ_42_ and tau levels were significantly higher for the Mayo participants. Dealing with these issues is essential for the appropriate use of these plasma biomarkers.

This study is descriptive in nature and hopefully will be an impetus to a more extensive study of the impact of ethnic/racial factors and the determination of the causes of these biomarker differences. There are several possible causes that relate to sociocultural determinants of health including the effect of systemic racism, neighborhood deprivation, nutrition, environmental exposure, medical comorbidities, and access to healthcare. The HABS-HD study has recently added the perception of racism scale to our battery. We are longitudinally assessing the impact of racism, and other sociocultural influences on biological factors related to the disproportionate risk for Alzheimer's in diverse communities.

The use of plasma biomarkers of cognitive decline is promising given their low cost, less invasive nature, and ready availability but as the current research shows factors such as ethnoracial effects must be considered to enhance accuracy and utility.

## Data Availability Statement

The original contributions presented in the study are publicly available. This data can be found at: University of North Texas Health Science Center (UNTHSC) Institute for Translational Research (ITR), https://apps.unthsc.edu/itr/researchers.

## Ethics Statement

The studies involving human participants were reviewed and approved by Institutional Review Board University of North Texas Health Science Center. The patients/participants provided their written informed consent to participate in this study.

## HABS-HD Study Team

Sid E. O'Bryant, Kristine Yaffe, Arthur Toga, Robert Rissman, and Leigh Johnson.

## HABS-HD Investigators

Meredith Braskie, Kevin King, James R. Hall, Melissa Petersen, Raymond Palmer, Robert Barber, Yonggang Shi, Fan Zhang, Rajesh Nandy, Roderick McColl, David Mason, Bradley Christian, Nicole Philips, Stephanie Large, and Rocky Vig.

## Author Contributions

JH and SO'B conceptualization and design of the study, acquisition, analysis, and interpretation of data, drafting and revising the manuscript, final approval of the version to be published, and agreement to be accountable for the accuracy and integrity of the work. MP, LJ, and SO'B: design of the study, acquisition and interpretation of data, drafting and revising the manuscript, final approval of the version to be published, and agreement to be accountable for the accuracy and integrity of the work. All authors contributed to the article and approved the submitted version.

## Funding

This work was supported by the National Institute on Aging of the National Institutes of Health under Award Numbers R01AG054073 and R01AG058533. This work was also supported in part by NIH/NIBIB award P41-EB015992.

## Author Disclaimer

The content is solely the responsibility of the authors and does not necessarily represent the official views of the National Institutes of Health.

## Conflict of Interest

SO'B has multiple patents on precision medicine for neurodegenerative diseases and is the founding scientist of Cx Precision Medicine. The remaining authors declare that the research was conducted in the absence of any commercial or financial relationships that could be construed as a potential conflict of interest.

## Publisher's Note

All claims expressed in this article are solely those of the authors and do not necessarily represent those of their affiliated organizations, or those of the publisher, the editors and the reviewers. Any product that may be evaluated in this article, or claim that may be made by its manufacturer, is not guaranteed or endorsed by the publisher.
